# De novo normotensive scleroderma renal crisis six years after living-donor renal transplantation in a patient with overlapping systemic sclerosis/systemic lupus erythematosus syndrome: a case report

**DOI:** 10.1186/s12882-023-03416-7

**Published:** 2023-12-04

**Authors:** Hajime Sanada, Satoshi Hara, Makoto Horita, Hiroyuki Kawahara, Misaki Yoshida, Yoshinori Takahashi, Shunsuke Tsuge, Takeshi Zoshima, Ryo Nishioka, Kiyoaki Ito, Ichiro Mizushima, Takashi Matsushita, Mitsuhiro Kawano

**Affiliations:** 1 Department of Rheumatology, Kanazawa University Hospital, 13-1, Takara-machi, Kanazawa, Ishikawa Japan; 2 Department of Dermatology, Kanazawa University Graduate School of Medical Sciences, Kanazawa, Japan

**Keywords:** Scleroderma renal crisis, Systemic sclerosis, Kidney transplantation

## Abstract

**Background:**

Scleroderma renal crisis (SRC) is a critical kidney involvement of systemic sclerosis (SSc), often resulting in end-stage renal disease. Although the recurrence of SRC in the allograft has been reported, the development of de novo SRC after kidney transplantation has not been reported. Furthermore, normotensive SRC, which rarely occurs, makes prompt diagnosis more challenging. This fact should be recognized widely among nephrologists.

**Case presentation:**

We report a 37-year-old Japanese man with overlapping SSc/systemic lupus erythematous syndrome who developed normotensive SRC in the transplanted kidney shortly after glucocorticoid escalation. Six years prior to admission, he underwent an ABO-compatible living donor kidney transplantation because of lupus nephritis. He was admitted to our hospital for gradually worsening kidney dysfunction. A kidney biopsy showed idiopathic granulomatous interstitial nephritis and high-dose prednisolone was prescribed. Although renal function improved tentatively, it deteriorated again a week later. A secondary kidney biopsy revealed acute thrombotic microangiopathy, leading to the diagnosis of normotensive SRC because all other causes were excluded, and blood pressure was within normal range. Adding an angiotensin-converting enzyme inhibitor and tapering glucocorticoid slowed the speed of deterioration of his kidney function, but he finally required hemodialysis induction.

**Conclusions:**

SRC can newly develop even in the transplanted kidney, especially when high-dose glucocorticoid is administered. Normotensive SRC makes the diagnosis challenging, so nephrologists should carefully monitor patients with SSc and transplanted kidneys to treat SRC promptly.

## Background

Systemic sclerosis (SSc) is an immune-mediated disease characterized by prominent fibrosis and vasculopathy [1]. Scleroderma renal crisis (SRC) is a life-threatening kidney involvement of SSc. It usually occurs rapidly with severe hypertension and progressive renal dysfunction, worsening the renal and overall prognosis [2]. Although SRC could relapse after renal transplantation, de novo SRC in the transplanted kidney has not been reported. Furthermore, normotensive cases, which rarely occur, could make it more challenging to recognize SRC promptly, leading to diagnostic and therapeutic delays [3].

Here, we report a case with overlapping SSc/systemic lupus erythematosus syndrome who newly developed normotensive SRC in a transplanted kidney shortly after glucocorticoid escalation. SRC should be kept in mind even after kidney transplantation when using high-dose glucocorticoid.

## Case presentation

A 36-year-old Japanese man was admitted to our hospital for worsening renal function. At the age of 17, he was diagnosed with diffuse cutaneous SSc with a positive anti-Scl-70 antibody. When he was 26-years-old, he developed proteinuria with positive anti-dsDNA antibody, hypocomplementemia, and polyarthritis. A kidney biopsy revealed class V lupus nephritis. He was treated with intensive immunosuppressive therapy of prednisolone (PSL) 40 mg/day (0.6 mg/kg/day) and tacrolimus (TAC) 3 mg/day; however, his renal function resulted in end-stage renal disease. At age 30-years-old, he underwent ABO-compatible kidney transplantation. The transplantation was successful, and he had been treated with PSL, mycophenolate mofetil (MMF), and TAC. However, he started suffering from cytomegalovirus (CMV) disease repeatedly. MMF was switched to everolimus (EVL) to control CMV disease 6 weeks prior to admission. His kidney function had been stable with serum creatinine (sCr) around 1.10 mg/dL but declined 2 weeks before admission.

On admission, he was asymptomatic. His medication included PSL 5 mg/day, TAC 5 mg/day, and EVL 1.5 mg/day. His blood pressure was 118/70 mmHg. Physical examination revealed fine crackles in the bilateral lungs, marked skin thickening extended to the trunk resulting in the fingers’ contracture. Multiple ulcers were observed on the tips of the fingers, elbows, and knees. Blood tests showed sCr 1.65 mg/dL, estimated glomerular filtration rate 40.1 ml/min/1.73m^2^, and anti-dsDNA antibody was negative. CMV antigenemia was not detected. The TAC and EVL troughs were 2.2 ng/mL and 4.4 ng/mL, respectively. Urine protein was 0.50 g/gCr. Granular casts were detected with no microscopic hematuria.

**Fig. 1:**
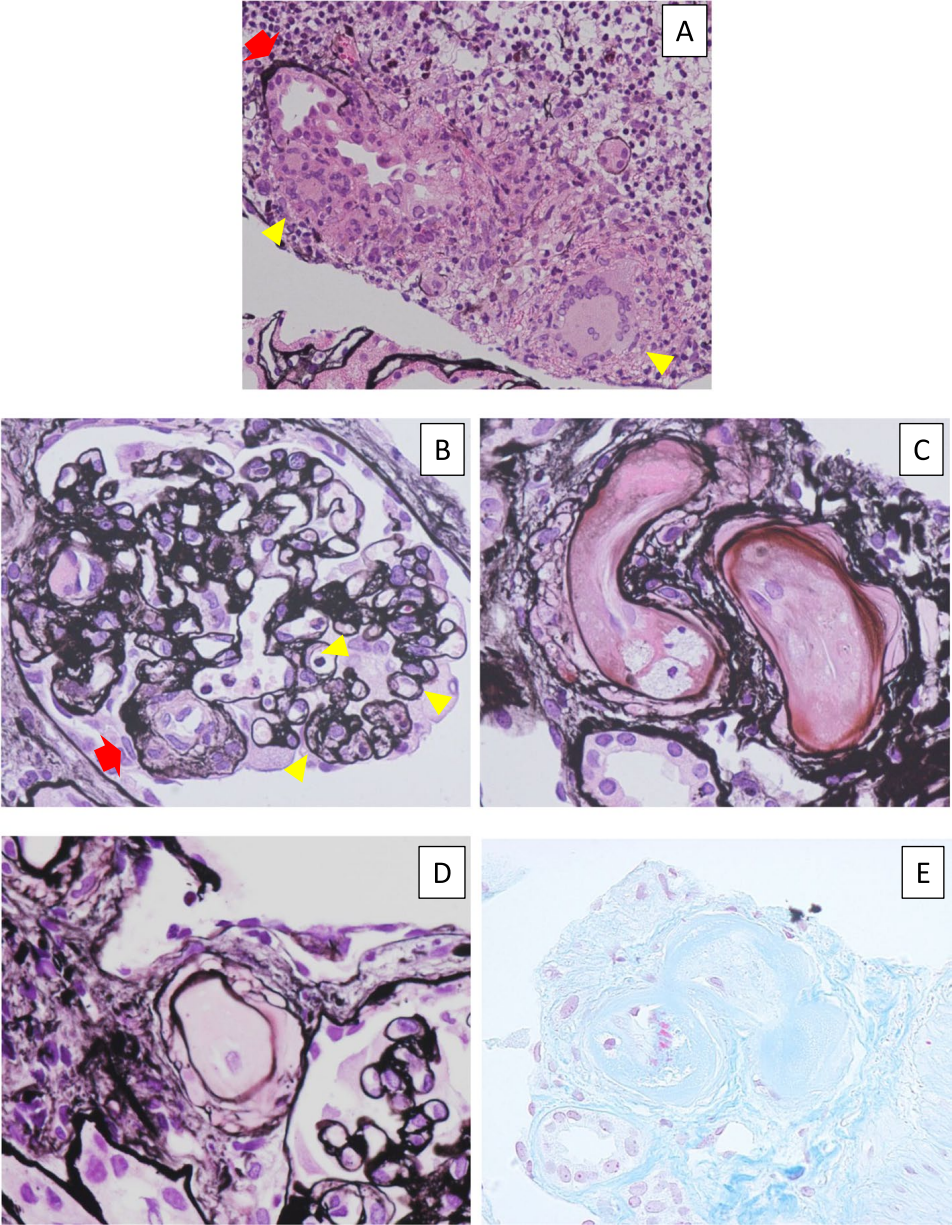
Representative pathological findings of the first and second kidney biopsies (**A** for the first biopsy, **B**-**E** for the second biopsy). **A** Focal interstitial mononuclear inflammatory cells infiltration was evident. Of note is that destructive tubulitis (arrow) was observed along with multinuclear giant cells (arrowheads). [Periodic acid-methenamine-silver (PAM) staining, × 100]. **B** A glomerulus showed double contours of the glomerular basement membrane (arrowheads) and microaneurysm (arrow) (PAM staining, × 400). **C**, **D** Vascular lumens were severely narrowed by mucoid intimal thickening of the arterioles. Arteriolar hyalinosis was also shown (PAM staining, × 400). **E** Thrombi were found in the arterioles (Masson-Trichrome staining, × 400)

**Fig. 2:**
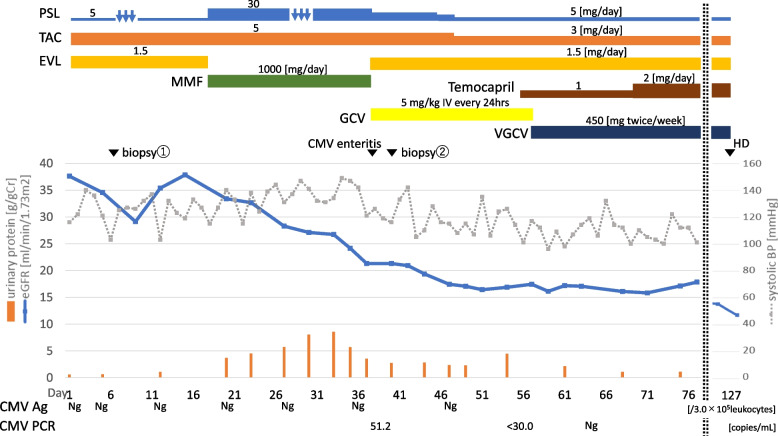
The clinical course. *PSL* prednisolone, *TAC* tacrolimus, *EVL* everolimus, *MMF* mycophenolate mofetil, *GCV* ganciclovir, *VGCV* valganciclovir, *CMV Ag* Cytomegalovirus antigenemia, *HD* Hemodialysis, *eGFR* estimated glomerular filtration rate, *BP* blood pressure, *Ng* negative

Renal biopsy showed focal destructive tubulitis and interstitial mononuclear cell infiltration with multinuclear giant cells (Fig. 1A), indicating granulomatous interstitial nephritis (GIN). Secondary causes for GIN such as drugs, viruses, tuberculosis, and sarcoidosis were not evident; he had not taken any medications including nonsteroidal anti-inflammatory drugs and antibiotics. He had no symptoms or serological and radiological abnormalities suggestive of viruses, tuberculosis, or sarcoidosis. Intravenous methylprednisolone 500 mg was administered, and PSL dose was increased to 30 mg/day (0.8 mg/kg/day). EVL was switched to MMF. His renal function ameliorated tentatively but worsened a week later (Fig. 2). His platelet counts decreased from 300,000 to 150,000/μL, while neither schistocytes nor low haptoglobin were observed. Three weeks after worsening renal function again, diarrhea appeared along with serum CMV-PCR 51.2 copies/mL, leading to the diagnosis of CMV enteritis. Although intravenous ganciclovir improved enteritis quickly, his renal function continued deteriorating and his platelet counts had not recovered; therefore, a second kidney biopsy was performed.

The specimen contained four glomeruli, one of which showed global sclerosis. All remaining glomeruli collapsed. A glomerulus showed double contours of the glomerular basement membrane and microaneurysm (Fig. 1B). Diffuse interstitial mononuclear cell infiltration and fibrosis were found, but multinuclear giant cells disappeared. Mucoid intimal thickening of the arteriole was conspicuous and microvascular thrombi were present in some areas (Fig. 1C-E). There was moderate fibrotic intimal thickening in the interlobular arteries and severe intimal hyalinosis in the arterioles. There was no evidence of vasculitis, including peritubular capillaritis. Immunofluorescence was all negative, including C4d. Electron microscopy was not performed due to sample errors. We diagnosed it as acute thrombotic microangiopathy (TMA). He showed no signs of antibody-mediated rejection, active lupus nephritis, thrombotic thrombocytopenic purpura (TTP), or hemolytic uremic syndrome (HUS). The TAC trough had been under 5 ng/mL. CMV enteritis had not occurred at the time renal deterioration appeared again. Therefore, we concluded his TMA as normotensive SRC, considering his blood pressure had not elevated. Temocapril 2 mg/day was added, and the deterioration of renal function stopped at about sCr 3.6 mg/dL (Fig. 2). However, his renal function continued to worsen gradually, leading to the initiation of hemodialysis 1 month later.

## Discussion and conclusions

We report a case of normotensive SRC newly developed after renal transplantation. His renal function had been stable for 6 years; however, PSL treatment for idiopathic GIN triggered acute TMA. After exclusion of various causes of TMA, we diagnosed it as SRC. To the best of our knowledge, de novo SRC in the transplanted kidney has not been reported and normal blood pressure could lead to the diagnostic delay of SRC, resulting in worse renal outcome.

SRC was the most likely cause of acute TMA in the present case. The incidence of de novo TMA after renal transplantation is 0.8―13.8%, and the major causes include drug toxicity such as calcineurin inhibitors (CNIs) and mammalian target of rapamycin inhibitors, antibody-mediated rejection, recurrent primary diseases, viral infections, TTP, and HUS [4]. Thus, the exclusion of these causes was mandatory to diagnose SRC in the present case. The effect of CNI toxicity could not eliminate because it could occur any time after transplantation, and CNI trough level sometimes does not correlate with the development of TMA [5, 6]. However, CNI toxicity would be unlikely to cause ‘acute’ TMA in our case because TAC trough had been kept lower than 5 ng/mL. Regarding the possibility of rejection, C4d immunostaining was negative, and no donor-specific antibody was detected, in addition to no histological findings of rejection. In addition, no signs or symptoms of active lupus nephritis or antiphospholipid antibody syndrome were noted. Considering CMV-TMA, no evidence of active CMV disease was found when renal function started deteriorating again. Since previously reported cases of CMV-TMA presented with more than one feature of CMV disease [7, 8], the course of this case did not fit as CMV-TMA. Finally, there were clinically no signs or symptoms of TTP and HUS, although we have not measured ADAMTS13. Therefore, we concluded that SRC caused rapidly progressive renal dysfunction because of acute TMA in the present case.

High-dose glucocorticoids under TAC treatment might trigger de novo SRC development in the present case. Newly developed SRC in a transplanted kidney has not been reported, while SRC recurrence has been reported in rare situations. Gibney et al. documented three (2.1%) of 142 kidney transplant cases of SSc patients had a relapse of SRC within 3 years [9]. Bertrand et al. reported that three (8.3%) of 36 transplanted kidneys occurred SRC recurrences between one and 57 months [10]. Pham PT et al. reviewed five cases of SRC recurrence in the transplanted kidneys between 1 month and 2 years [11]. Risk factors for the recurrence of SRC after transplantation have been unidentified, while those for SRC development in native kidneys have been confirmed, including early, diffuse cutaneous SSc, anti-RNA polymerase III antibody, high-dose glucocorticoids (≥ 15 mg/day), rapid progression of skin thickening, and tendon friction rubs [2]. One case series of SRC recurrence showed that new onset anemia, worsening pericardial effusion, and progression of diffuse skin thickening preceded SRC [11], suggesting that risk factors predictive for SRC recurrence might be similar to those for new SRC development. In addition, several case reports suggest that CNIs trigger SRC development even under low trough levels [12], raising the possibility of TAC treatment as the trigger of SRC development. Considering the clinical course that acute TMA developed immediately after increasing PSL dose, high-dose glucocorticoid under TAC treatment might cause the development of SRC in the present case. Although the present case did not present SRC at the time of treatment for lupus nephritis, a relatively higher dose of glucocorticoid might be associated with SRC development because glucocorticoid-induced SRC has developed in cases receiving high doses [2, 13, 14]. Taken together, SSc patients with transplanted kidneys who take high-dose PSL under CNIs treatment should be monitored carefully. Further studies are needed to clarify the risk factors for SRC development in transplanted kidneys, including PSL and CNI doses.

Another notable point of our case is that SRC was normotensive. SRC without hypertension has been observed in approximately 10% of SRC cases [3]. There are several hypotheses of why some SRC patients are normotensive. Firstly, Steen VD et al. noted that blood pressure elevates but does not reach 140 mmHg in some SRC due to lower baseline of blood pressure [15]. Secondly, a research group recently proposed a classification of the pathogenesis of SRC as narrowly defined SRC (nd-SRC) and SSc-TMA [16]. Nd-SRC refers to the intimal thickening of injured arcuate and interlobular arteries, which reduces renal blood flow and activates the renin-angiotensin system, resulting in the typical course of SRC that shows acute onset renal failure accompanied by marked hypertension. On the contrast, SSc-TMA refers to the injury of arterioles and capillaries, which contributes to gradual kidney dysfunction and thrombocytopenia without increasing blood pressure. These two conditions could overlap in varying degrees, and a patient could be normotensive when SSc-TMA was dominant. In our case, endothelial cell injury in arterioles and capillaries supports the hypothesis that SSc-TMA contributed to the pathogenesis of normotensive SRC. Normotensive SRC should be considered when SSc patients presented progressive kidney dysfunction with acute TMA after careful examination to exclude other causes of acute TMA in the kidney allografts.

In conclusion, we reported the case of normotensive SRC newly developed in a transplanted kidney shortly after glucocorticoid escalation. SRC can develop anytime in SSc patients, and it cannot be ruled out only based on normal blood pressure.

## Data Availability

All the data relevant to this report are included in the manuscript.

## Abbreviations

SRC: Scleroderma renal crisis
SSc: Systemic sclerosis
PSL: Prednisolone
MMF: Mycophenolate mofetil
TAC: Tacrolimus
CMV: Cytomegalovirus
EVL: Everolimus
sCr: Serum creatinine
GIN: Granulomatous interstitial nephritis
TMA: Thrombotic microangiopathy
TTP: Thrombotic thrombocytopenic purpura
HUS: Hemolytic uremic syndrome
CNIs: Calcineurin inhibitors
